# Naringenin and Hesperidin as Promising Alternatives for Prevention and Co-Adjuvant Therapy for Breast Cancer

**DOI:** 10.3390/antiox12030586

**Published:** 2023-02-27

**Authors:** Maria Beatriz Madureira, Virginia Marcia Concato, Ellen Mayara Souza Cruz, Juliana Maria Bitencourt de Morais, Fabricio Seidy Ribeiro Inoue, Natália Concimo Santos, Manoela Daniele Gonçalves, Milena Cremer de Souza, Thalita Basso Scandolara, Mariane Fontana Mezoni, Murilo Galvani, Fábio Rodrigues Ferreira Seiva, Carolina Panis, Milena Menegazzo Miranda-Sapla, Wander Rogério Pavanelli

**Affiliations:** 1 Laboratory of Immunoparasitology of Neglected Diseases and Cancer, State University of Londrina, Londrina 86057-970, Brazil; 2 Department of Biology, Biological Sciences Center, State University of Northern Paraná—UENP, Luiz Meneghel Campus, Bandeirantes 86360-000, Brazil; 3 Laboratory of Biotransformation and Phytochemical, State University of Londrina, Londrina 86057-970, Brazil; 4 Department of Pathological Sciences, State University of Londrina, Londrina 86057-970, Brazil; 5 Laboratory of Tumor Biology, State University of West Paraná, Unioeste, Francisco Beltrão 85601-080, Brazil; 6 Department of Genetics, Federal University of Rio de Janeiro—UFRJ, Rio de Janeiro 21941-901, Brazil; 7 Postgraduate Program in Pharmaceutical Science, University of Vale do Itajaí, Itajaí 88302-901, Brazil

**Keywords:** citrus fruits, bioactive compounds, breast cancer, flavanones, naringenin, hesperidin, antioxidants, anticancer activity

## Abstract

Citrus (genus *Citrus* L.) fruits are essential sources of bioactive compounds with antioxidant properties, such as flavonoids. These polyphenolic compounds are divided into subclasses, in which flavanones are the most prominent. Among them, naringenin and hesperidin are emerging compounds with anticancer potential, especially for breast cancer (BC). Several mechanisms have been proposed, including the modulation of epigenetics, estrogen signaling, induction of cell death via regulation of apoptotic signaling pathways, and inhibition of tumor invasion and metastasis. However, this information is sparse in the literature and needs to be brought together to provide an overview of how naringenin and hesperidin can serve as therapeutic tools for drug development and as a successful co-adjuvant strategy against BC. This review detailed such mechanisms in this context and highlighted how naringenin and hesperidin could interfere in BC carcinogenesis and be helpful as potential alternative therapeutic sources for breast cancer treatment.

## 1. Introduction

Breast cancer (BC) is the most common malignancy in women worldwide and the leading cause of cancer-related deaths in the population [1]. BC cases are expected to increase by 4.4 million annually by 2070 [2]. Based on the expression of hormonal receptors (estrogen—ER and progesterone—PR) and the human epidermal growth factor receptor 2 (HER2) amplification, BC is classified as luminal (ER and/or PR+), HER2-amplified (any ER/PR status), or triple-negative (ER and PR -). These molecular subtypes are pivotal for clinical management and chosen therapeutic strategies [3,4]. BC is a multifactorial disease, and despite advances in screening and treatment, the underlying mechanisms and treatment alternatives are under continuous investigation [5].

Oxidative stress has been implicated as a mechanism involved in breast cancer development [6,7]. This type of stress can result from various factors, including menopause, aging, exposure to estrogen, or even genetic predisposition, and occurs when there is an imbalance between the production and neutralization of reactive species (RS). Normal cells continuously generate RS from the incomplete oxygen reduction that occurs during respiratory chain reactions. Thus, complex antioxidant systems are essential to protect the organism and are composed of a range of enzymatic (such as superoxide dismutase (SOD), glutathione peroxidase (GPx), glutathione reductase, catalase), and nonenzymatic antioxidants (e.g., glutathione (GSH), vitamins C and D), which help to reduce or inhibit oxidative damage caused by RS [6,8,9,10]. These systems act as scavengers or quenchers of RS, converting these reactive species into less reactive intermediates and preventing cell damage [11,12]. Antioxidants significantly prevent or delay the oxidation of sensitive substrates, such as lipids, proteins, and DNA, even at low concentrations, thereby maintaining cellular homeostasis [13].

However, when the oxidant-antioxidant balance is disrupted due to excessive RS production or insufficient antioxidants, the accumulation of RS can cause oxidative stress. This condition is directly linked to the physiopathology of numerous diseases, including chronic inflammation and cancer. Despite significant redundancy in the antioxidant systems, they all neutralize RS to preserve redox stability and protect lipids, proteins, and DNA from oxidative stress damage [14,15]. Under sustained environmental stress, RS can cause significant damage to cell structures, including DNA damage, which can contribute to abnormal cell growth and promote metastasis, angiogenesis, and hypoxia adaptation [16]. DNA damage can lead to genomic instability, which is a necessary step for cancer initiation, promotion, and progression [10,17,18].

Nonenzymatic antioxidants can be obtained from the diet and are indispensable for proper defense against widespread oxidation [19]. Therefore, they play a crucial role in maintaining cellular health, and maintaining an antioxidant-rich diet has been shown to prevent more than two-thirds of human cancers [14,20]. It is widely accepted that fruits and vegetables rich in antioxidants are pivotal components of a healthy diet and can reduce the incidence of numerous malignancies [14]. Phytochemicals appear to contribute to cancer prevention by reversing the malignant transformation caused by oxidative stress, indicating their chemopreventive potential [21]. It is worth noting that phytochemicals are great sources of oncological drugs and are usually cost-effective [22].

Investigating natural compounds derived from vegetables and fruits has the potential to provide new insights into both prevention and complementary therapeutics, thereby strengthening the field of “green chemistry.” Among the fruits commonly consumed worldwide, citrus fruits belong to the *Citrus* genus, which encompasses some of the most widely cultivated fruit crops worldwide and stands out as a rich source of phenolic compounds that have been linked to reducing oxidative stress-related disorders [16,23]. The phenolic compounds found in citrus fruits have been shown to have several positive impacts on the body, including reducing inflammation [24], improving cardiovascular health [25], and protecting against oxidative stress-related disorders [26,27]. Several in vitro and in vivo studies have shown that flavonoids, especially flavanones, the main class of flavonoids in citrus extracts, such as naringin and naringenin [22,28], possess antiproliferative, anti-inflammatory, and pro-apoptotic properties [29,30,31,32,33,34]. Other compounds found in citrus fruits that have demonstrated potential anticancer effects include quercetin [15,35,36,37,38,39,40,41], hesperidin [42,43,44], hesperetin [45,46], polymethoxyflavones [47,48,49], eriodyctiol [50,51], bergapten [52,53], tangeretin [54,55], auraptene [56,57,58], limonin [59], naringenin [60,61,62,63,64,65], and naringin [66,67,68], as shown in (Table 1). These findings suggest a potential role for flavonoids in cancer therapy, including breast cancer.

Citrus fruits are an important source of phenolic compounds that have the properties and the potential to be co-administered in chemotherapeutic regimens, but their mechanism of action is complex and requires further research. While there is evidence suggesting that citrus flavones may have a protective effect against breast cancer, more research is needed to fully evaluate the potential for their use in breast cancer prevention or treatment. In this context, the present review focuses on the current understanding of the anti-breast cancer effects of naringenin and hesperidin to investigate potential insights for co-adjuvant treatment strategies.

## 2. Data Analysis Methodology

This review focuses on research considering the composition, function, and anti-cancer properties of two citrus flavanones, naringenin and hesperidin, for BC. PubMed, ScienceDirect, and Google Scholar databases were searched using the keywords “citrus fruits/naringenin/anti-breast cancer”, “Citrus fruits/hesperidin/anti-breast cancer”, “naringenin/anti-breast cancer”, and “hesperidin/anti-breast cancer”. The studies selected were published between 2012 and 2022, and a total of 1.861 articles were analyzed thoroughly, examining the titles and abstracts to verify their relevance. We selected 162 original articles that specifically analyzed the bioactivity of naringenin and hesperidin concerning BC. Articles that did not fit these criteria were excluded.

## 3. Naringenin and Hesperidin: An Overview

### 3.1. Citrus Fruits and Flavanones

The genus *Citrus*, a member of the Rutaceae family and the Aurantioidae subfamily, is one of the most widely cultivated and consumed plant species globally [23,69]. Originating in the Himalayan region of southwestern China, northeastern India, and northern Burma, it has since been grown in over 140 countries [70]. The taxonomy of the genus *Citrus* is complex and controversial, mainly because of sexual compatibility between species and genera, the high frequency of bud mutations, and the long history of cultivation and wide dispersion, making the quantification of species uncertain, but it is known that this genus contains numerous species that differ in their fruit, flower, leaf, and twig characteristics [71].

Some of the most commercially important species of *Citrus* include the sweet orange (*Citrus sinensis*), sour orange (*C. aurantium*), mandarin (*C. reticulata*), grapefruit (*C. paradisi*), pummelo (*C. grandis*), lemon (*C. limon*), citron (*C. medica*), lime (*C. aurantifolia*), kumquat (*C. japonica*), and hybrids [70]. Citrus fruits are rich in secondary metabolites such as polyphenols and terpenoids [71]. A hundred polyphenols have been detected in citrus, with flavonoids being the most important bioactive components with a wide variety and distribution present in almost all the parts of citrus fruits in different species [72].

**Figure 1:**
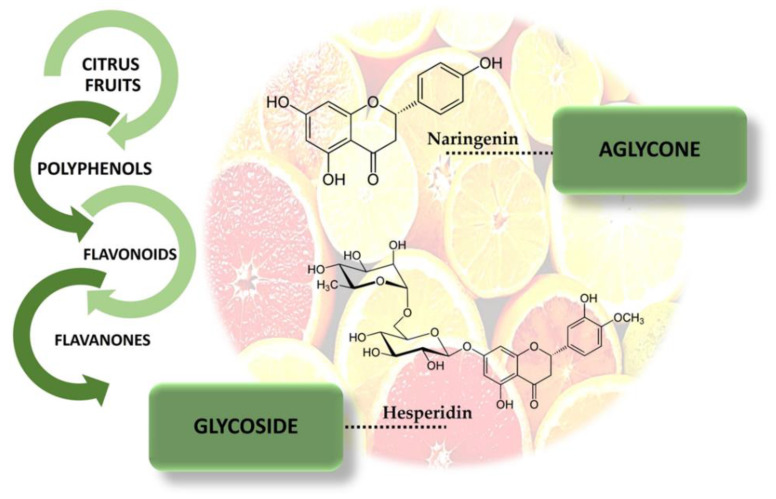
Naringenin and hesperidin derived from citrus fruits. Schematic representation of the sequential distribution of the major functional bioactive compounds (polyphenols, flavonoids, and flavanones) found in *Citrus* species and characterization of the molecular structure of the flavanone subclasses aglycone (naringenin) and glycoside (hesperidin).

Flavonoids, responsible for the flavor and color of fruits and flowers, are involved in metabolic processes and chemical signaling. They are further divided into subclasses such as flavanones, flavonols, anthocyanins, flavones, and polymethoxyflavones [73,74]. Although the content and types of flavonoids vary among *Citrus* species and fruit parts, flavanones are the most important in *Citrus* species, which are represented by two main categories, further classified into glycoside (hesperidin and narirutin) or aglycone (hesperetin and naringenin) (Figure 1) [69,71,73]. They can be found in all plant parts, such as stems, branches, bark, flowers, leaves, roots, and seeds [71].

Of all the flavanone varieties, hesperidin (3,5,7-trihydroxyflavanone 7-rhamnoglucoside) and naringenin (4′,5,7-trihydroxyflavanone) are the predominant flavanones in citrus fruits [75,76] and can be found in all parts of the plant, including stem, branches, bark, flowers, leaves, roots, rhizomes, seeds, fruits, and peels [71]. These flavones have well-established beneficial effects on human health and, in addition to citrus fruits, can also be found in other natural sources such as honey, mint, and tomatoes [76].

Hesperidin is a flavanone glycoside consisting of hesperetin (aglycone) and rutinose disaccharide (glucose-related rhamnose) (Table 1). It is most abundant in clementines, sweet oranges, mandarin oranges, and lemons. Studies have shown that hesperidin is most abundant in the peel and membranous sections of citrus fruits [72]. Naringenin is the predominant flavanone found mainly in grapefruit. It is an aglycone flavanone, and it can exist in different forms depending on the sugar molecule attached to it. Naringenin can be found as glycoside forms naringenin-7-*O*-rutinoside (narirutin) and naringenin-7-*O*-glucoside (naringin), both occurring naturally as aglycone and glycoside forms [72,77].

In general, the biological properties of hesperidin and naringenin include antioxidant, anti-inflammatory, inhibitory effects against obesity-associated diseases, and anti-cancer properties. They also act in cardiovascular protection and analgesic manner [30,43,78,79,80,81]. Moreover, studies have demonstrated that these compounds can modulate molecular targets and signaling pathways involved in cell survival, proliferation, differentiation, migration, angiogenesis, and hormonal activity [82].

### 3.2. Sites of Interaction and Structure-Activity Relationship by Naringenin and Hesperidin

**Figure 2:**
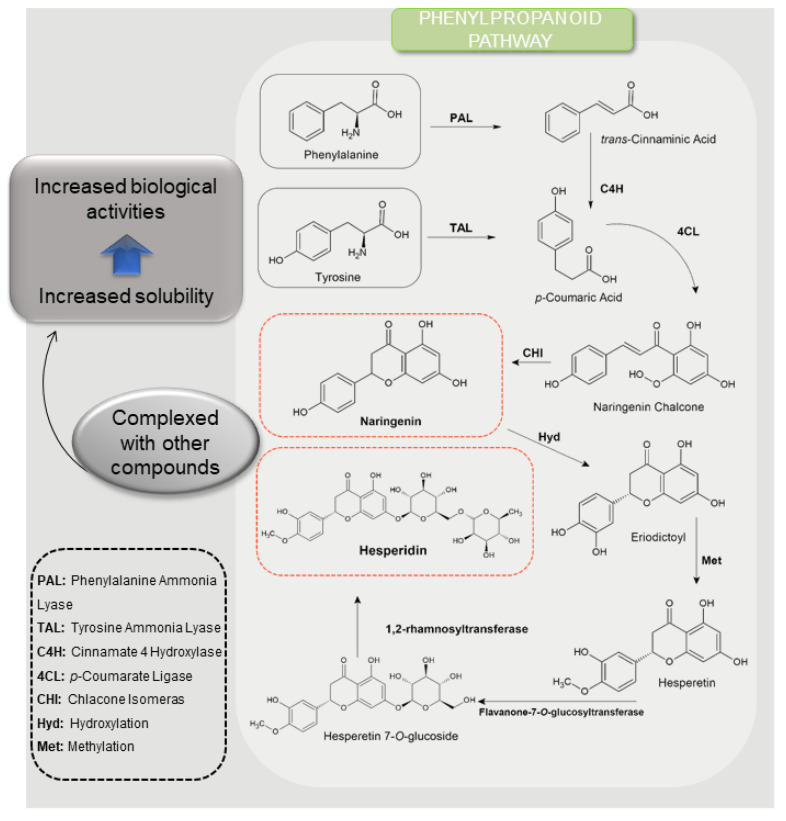
Naringenin and hesperidin follow a common pathway through the phenylpropanoid pathway.

Secondary metabolites are generated during the biosynthesis process, which, for naringenin and hesperidin, follow a common pathway through the phenylpropanoid pathway. First, the phenylalanine is transformed into *p-*coumaronyl-CoA through the action of the enzymes phenylalanine ammonia-lyase (FAL), cinnamate 4-hydroxylase (C4H), and 4-coumaronyl-CoA ligase (4CL). Then, three malonyl-CoA molecules combine with one *p*-coumaronyl-CoA to form an aromatic ketone converted to naringenin (Figure 2). Subsequent events of hydroxylation and methylation result produce hesperidin [74]. Actinomycetes can also make naringenin. The bacterium *Streptomyces clavuligerus* synthesizes naringenin using *p-*coumaric acid and the P450 monooxygenase enzyme as pathway initiators rather than the general phenylpropanoid pathway seen in plants. Other bacteria in the genus *Streptomyces* can produce naringenin by the same principle, using *p*-coumaric acid or other pathway initiators, such as caffeic acid and benzoic acid [83].

The basic structure of phenolic compounds is based on two benzene rings and fifteen carbon atoms linked by a short chain of three carbon atoms, which in turn form a pyran ring. Structural variations are currently used to classify different types of flavonoids, such as the content of hydroxyl and methoxyl groups [84]. This is the case for flavanones, a phenolic class including naringenin and hesperidin. These compounds have a saturated C ring, and due to this fact, the double bonds present on carbons 1 and 2 are also saturated [73].

Naringenin is a solid compound with dissociation constants (pKa) values of 7.05 and 8.84, with a melting point of 208–251 °C, and basic nature. The compound is soluble in ethanol, dimethylformaldehyde dimethylsulfoxide, but poorly soluble in water (4.38 μg/mL). In a similar way, hesperidin also shows a low solubility (4.95 μg/mL) [85]. This characteristic of the two flavanones means that their biological activities are reduced when used alone. Therefore, the use of other compounds complexed to naringenin and hesperidin may be an alternative to increase their solubility in an aqueous medium and their biological activity. Cyclodextrin and its derivatives are the most used compounds for the formation of this type of complex. For example, naringenin complexed with hydroxylpropyl-β-cyclodextrin (P-β-CD) achieves a solubility of >500 g/L at 20 °C [73,86].

A study examined the complexation of naringenin with different cyclodextrin derivatives, including β-cyclodextrin (β-CD), 2,6-di-O-methyl-β-cyclodextrin (DM-β-CD), and randomly methylated β-CD (RAMEB). The study found that the naringenin/RAMEB complex had increased stability and solubility in aqueous solutions, with stability constants of 1015.5 Kc (M-1). The main type of force involved in binding the complexes was found to be van der Waals, which had a binding energy six times higher than electrostatic forces in all of the complexes. Additionally, the naringenin/DM-β-CD complex was found to have a stronger cytotoxic effect on MCF-7 and HeLa cells than on free naringenin. The study also showed that hesperetin, the compound from which hesperidin is derived, had improved stability and solubility after complex formation, as well as increased cytotoxicity, similar to naringenin [86].

Studies exploring the molecular interactions of naringenin and hesperidin are limited. Those examining the molecular interactions of hesperidin are even more scarce. In our search, we found a few papers that evaluated derivatives of this compound. One interaction that has been discovered for naringenin is with lysozyme, where it acts as a non-competitive inhibitor of the enzyme by binding to its active site through the remnants of the amino acids tryptophan (Trp) 62, 63, and 108. This binding uses hydrophobic interactions, and positive entropy change (ΔS°) values contribute to the binding reaction. These findings, in a model using *Micrococcus lysodeikticus*, suggest that naringenin may act as an inhibitor of the lysozyme molecule [87]. Hesperidin has also shown the ability to spontaneously interact with the active site of trypsin to form a flavonoid-trypsin complex. This type of interaction influences the hydrophobicity of the microenvironment of tryptophan (Trp) residues, leading to a decrease in the enzymatic activity of trypsin [88].

Similarly, hesperidin is also able to inhibit the enzyme xanthine oxidase (XO), an essential enzyme of the purine catabolism pathway indirectly associated with pathological conditions such as cancer. Six products selected based on docking simulation studies were synthesized as aniline and hydrazine derivatives 3HDa 1–3 and 4HDb 1–3. The compounds showed potential antioxidant activity in vitro and an inhibitory effect on XO capacity in a competitive manner, with IC_50_ ranging from 0.263 μM–14.870 μM. The molecular simulation verified that the compounds showed interaction with the amino acid residues phenylalanine 798 (Phe798), glutamine 1194 (Gln1194), arginine 912 (Arg912), threonine 585 (Thr585), serine 1080 (Ser1080), and methionine 1038 (Met1038) positioned within the XO binding site [89].

The pharmacological mechanisms of neohesperidin dihydrochalcone (NHDC), a commercially synthesized by the catalytic alkali hydrogenation of hesperidin [90], were evaluated in vivo and identified 19 metabolites, with 18 being characterized for the first time. The metabolic reactions were evaluated using an optimized liquid chromatography method. The study also used network pharmacology to determine the targets of the NHDC metabolites and found they were involved in various pathways related to cancer, ovarian steroidogenesis, proteoglycans in cancer, PI3K/protein kinase B (Akt) signaling pathway, and progesterone-mediated oocyte maturation, providing new insights into the pharmacological antitumoral potential mechanisms of NHDC [91].

The antioxidant properties of phytochemicals are particularly well-studied in cancer, as exacerbated free radical production is directly associated with developing malignant tumors [84]. The flavonoid antioxidant activity is linked to their chemical structure, i.e., the neutralizing free radicals’ properties are influenced by the arrangement, number, and shape of hydroxyl groups and the presence of glycosides. The higher the number of hydroxyl groups, the greater the compound’s antioxidant activity [74]. In this context, the loss of the hydroxyl group on carbon 5 of the naringenin (liquiritigenin) molecule increases its IC_50_ from 1.97 μM to 6.55 μM, and the presence of C(2)=C(3) double bond in the C ring of the (apigenin) molecule decreases the antioxidant capacity of the molecule when compared to naringenin (C(2)–C(3)) [92].

These findings show that naringenin and hesperidin have relevant properties. These compounds act on key enzymes in signaling pathways linked to inflammation and have evidence of potent antioxidant action. However, studies on naringenin and hesperidin and their molecular interactions still need to be made available. Still, these interactions are directly linked to their chemical structure and the type of bond between them. Even though it lacks further studies on their interaction and action mechanisms, naringenin and hesperidin may be an option for treating diseases such as cancers.

### 3.3. Anti-Breast Cancer Role of the Citrus-Derivated Compounds Naringenin and Hesperidin

Breast cancer, like other types of cancer, can be initiated and progressed by endogenous or exogenous oxidative stress, which can also increase therapy resistance, angiogenesis, and metastasis [7,93]. In this context, several studies have shown the anti-breast cancer role of naringenin. Naringenin cytotoxic effects were evaluated against three cell lines, including MDA-MB-231 and MDA-MB-468, both of which are triple-negative breast cancer cell lines, and CHO, a Chinese hamster ovary cell line, in comparison to kaempferol. Naringenin showed cytotoxicity against MDA-MB-468 and MDA-MB-231, obtaining IC_50_ values of 238 μg/mL and 70 μg/mL, respectively, without causing toxicity to CHO cells. The combination of kaempferol and naringenin resulted in higher IC_50_ values of 43 μg/mL and 44 μg/mL against MDA-MB-468 and MDA-MB-231, respectively. Additionally, naringenin induced morphological changes in tumor cells while being non-toxic to normal cells [94].

The breast cancer resistance protein (BCRP), a critical ATP-binding cassette (ABC) efflux transporter, acts in drug and xenotoxin disposition; its overexpression in tumors can result in multidrug resistance (MDR). The antiproliferative activity of 99 flavonoids, which are major components of traditional Chinese medicine (TCM), vegetables, and fruits, were evaluated in the BCRP-MDCKII cell line (canine kidney cell line containing breast cancer resistance protein) in the presence of mitoxantrone. Of the 99 compounds tested, 11 showed more than 50% inhibition of cell viability, including naringenin. In the same study, it was suggested that naringenin might have potential as an adjunctive therapy for brain tumors since it increased the concentration of mitoxantrone and increased the cytotoxicity of doxorubicin and temozolomide in several cell lines of human brain tumors after rats received a single dose of 30 mg/kg naringenin [95].

Another study investigated whether naringenin would act on the E0771 (mammary adipose tissue carcinoma) cell line. Naringenin treatment inhibited cell proliferation, increased phosphorylation of AMP-activated protein kinase (AMPK), negatively regulated cyclin D1 expression, and induced cell death. To confirm these data, obese ovariectomized C57BL/6 mice were fed a high-fat (HF), high-fat low-naringenin diet (LN; 1% naringenin), or high-fat high-naringenin diet (HN; 3% naringenin) and xenografted with E0771 cells for three weeks. The authors observed more significant naringenin accumulation in the tumor than in the mammary adipose tissue in HN mice. Furthermore, NH decreased body weight, fat mass, adipocyte size, smooth muscle actin mRNA in mammary adipose tissue, and inflammatory cytokine. Also, compared to mice fed a HF diet, HN slowed tumor growth early but did not alter the final tumor weight, suggesting that naringenin exhibits beneficial effects on metabolic health and tumor origin [96].

In a study, ethanol extracts were obtained from the peels of several citrus fruits (*Citrus sinensis*, *C. aurantifolia*, *C. tangerine*, *C. aurantium*, *C. aurantium*, and *C. paradisi*), and their main components were isolated and tested for cytotoxicity against human breast cancer (MCF-7 and T47D) and normal human melanocytes (HFB4) cell lines. The results showed that the extracts and isolated compounds reduced cell viability without causing toxicity to normal cells, with naringenin being one of the most potent. The authors concluded that the effect of naringenin was not related to the modulation of the estrogen receptor or inhibition of aromatase. Furthermore, treatment with naringenin showed no uterotrophic activity and no changes in uterine weight or cornification, indicating that it does not have estrogenic activity. The treatment also reduced tumor volume and aromatase levels in mice with Ehrlich ascites carcinoma, suggesting that naringenin may have a potential role in breast cancer treatment before and after menopause. In contrast, hesperidin did not show significant anticancer activity at the tested concentration (0–50 µg/mL) in both cell lines [31].

Hesperidin also has pharmacological activity on breast cancer due to its anti-inflammatory and antioxidant properties [42]. Thus, the protective effect against oxidative stress and inflammation of hesperidin was evaluated in a study using MCF-7 cells and male Balb/c mice. The authors demonstrated that hesperidin can reduce cell proliferation starting at 40 µM. There was also reduced colony formation, increased nuclear condensation, and formation of apoptotic features. In the same study, mice treated with hesperidin showed an increased anti-inflammatory response, reducing IL-33 and TNF-α after stimulation with lipopolysaccharide (LPS). In addition, hesperidin treatment reduced lipid peroxidation and increased antioxidant capacity, where levels of the enzymes CAT and GSH increased in mice co-treated with LPS and hesperidin. They suggested that hesperidin may be a promising treatment for cancer [97].

**Figure 3:**
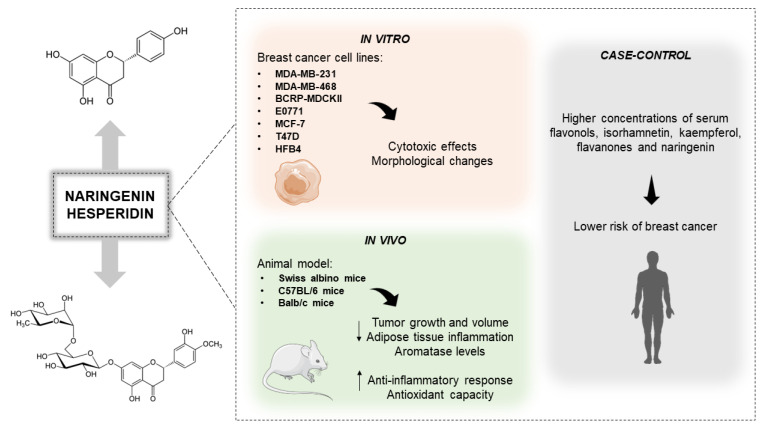
Schematic depiction of antitumor activity of naringenin and hesperidin.

In a case-control study, associations were made between serum concentrations of flavonols (quercetin, isorhamnetin, and kaempferol), flavones (apigenin and luteolin), flavanones (naringenin and hesperidin), and flavan-3-ols (catechin, epicatechin (EC), epigallocatechin (EGC), epicatechin-3-gallate (ECG), and epigallocatechin-3-gallate (EGCG)) and the risk of breast cancer in 792 female patients. It was demonstrated that higher blood levels of isorhamnetin, kaempferol, flavanones, and naringenin were associated with a lower risk of breast cancer (Figure 3) [98].

### 3.4. Naringenin and Hesperidin on Modulation of Epigenetics and Estrogens Mechanisms

Epigenetic modifications coordinate gene expression and interfere with hormonal signaling pathways, triggering multistep breast carcinogenesis [99]. Flavanones are involved in the epigenetic regulation of cancer pathogenesis by interfering with DNA methylation, histone modification, and expression of non-coding RNAs, events that influence tumor progression and drug resistance [100,101,102,103]. Both transcriptional receptor α (Erα or ER66), which stimulates cell proliferation, and transcriptional receptor β (Erβ), which facilitates cytostatic and differentiation processes, are involved in breast cancer progression [104,105]. Some of these epigenetic mechanisms lead to this abnormal ER activity, resulting in the upregulation of oncogenes, gene suppression, or the silencing of DNA repair genes [105].

One of the most described processes of DNA methylation in breast cancer occurs through the silencing of Wnt antagonist genes, leading to the constitutive activation of β-catenin and promoting stem cell renewal and proliferation [106]. The Wnt/β-catenin signaling pathway such as PI3K/AKT, p53, and MAPK have frequently altered signaling pathways in resistant tumor cells and transmit extracellular and intracellular signals involved in cell growth, proliferation, survival, differentiation, migration, metabolism, and apoptosis.

Flavonoids are a promising group of compounds with potential therapeutic applications for breast cancer. Their ability to regulate epigenetic modifications and interfere with hormonal signaling pathways makes them attractive candidates for the development of new treatments or the enhancement of existing therapies. Several studies have demonstrated the effectiveness of various flavonoids in inhibiting breast cancer cell proliferation, inducing apoptosis, and inhibiting the activity of estrogen receptors. Flavonoids have been reported to activate proapoptotic proteins such as the Bcl2-associated X protein (Bax), bH3-interacting death domain (Bid), and Bcl-2-interacting protein (Bim), and inhibit the anti-apoptotic members Bcl-2, Bcl2-like protein (Bcl2L), and the long isoform of Bcl-2-related protein (BclXL), making them potentially useful anticancer agents [107].

Breast cancer subtypes expressing hormone receptors (ER and/or progesterone receptor (PR)) are the most prevalent [93]. Interestingly, co-exposure to tamoxifen and naringenin was able to modulate four ER subtypes, downregulating mRNA transcription of ER66, ER36, and GPR30 but upregulating Erβ expression, suggesting an apoptosis induction process [108]. While targeting the ER with drugs like tamoxifen (Tam) is a common treatment approach, long-term use can lead to resistance. In combination with tamoxifen, naringenin (Nar-Tam) was found to be more effective at impairing the cell viability of MCF-7 than either treatment alone [109]. This is because naringenin inhibits proliferation pathways PI3K and MAPK activated in breast cancer cells, blocks the activation of ER, and prevents MCF-7 proliferation [109]. Even in the absence of estrogen, naringenin was shown to inhibit ERK1/2 phosphorylation and alter ERα localization, confirming that it affects signaling pathways other than those dependent on estrogen [110].

Due to their structural similarity to estrogen, they can also be referred to as phytoestrogens because they can modulate estrogen function. These compounds have the potential to act as selective estrogen receptor modulators (SERMs) and act as ERα antagonists, impacting hormone signaling and synthesis [111]. Naringenin has also been identified as a potential therapeutic target for inhibiting breast cancer stem cells (BCSC). Bioinformatics analysis and in vitro modeling showed that naringenin upregulates ERα and p53, which regulate transforming growth factor-β (TGF-β) and Wnt/β-catenin pathways, resulting in BCSC inhibition [112]. Reinforcing this study, Pang et al. [113] also showed a virtual screening descriptor model that investigated, through a luciferase reporter gene assay on the MCF-7 cell line, the effects of naringenin as a potential ERα antagonist [113].

In another study, the effect of naringenin, 17-estradiol (E2), and genistein on the activity of estrogen receptor (ER) in T47D-KBluc (cells containing the triplet reporter gene ERE (estrogen-responsive elements)-promoter-luciferase) and ER-negative MDA-MB-231 breast cancer cell lines was investigated. Naringenin was found to be a partial agonist (functioning as a competitive antagonist in the presence of a full agonist such as E2 or genistein) and not an efficient antagonist of the ER [114]. Additionally, in co-exposure with bisphenol A (BPA), naringenin was found to have a proapoptotic effect, which reduced the number of cells in both MCF-7 and T47D cell lines. On the other hand, BPA and E2 increased the number of cells in both cell lines by activating the Akt signaling pathway through Erα, leading to impaired cell proliferation and survival. However, naringenin prevented the proliferative effects of BPA by impairing Erα-mediated signals (Akt phosphorylation and Bcl-2 accumulation) and inducing persistent activation of p38, which initiated a proapoptotic cascade. Consequently, this study suggested that natural xenoestrogens, like naringenin, act as selective ER modulators by functioning on extranuclear Erα signaling pathways and providing critical information to develop tissue-specific E2 agonists and antagonists for breast cancer treatment [115].

The combination of hesperidin and chlorogenic acid also showed promising results for adjunctive therapies in breast cancer. The association enhanced toxicity towards MCF-7 cells but did not cause a cytotoxic effect on MCF-10A (non-tumorigenic epithelial). The synergistic effects of hesperidin and chlorogenic acid, which regulate multiple biochemical pathways, disrupt oxidative phosphorylation, mitochondrial dysfunction, and down-regulated synthesis of ATP and lipid functions by the ER pathway. The combined treatment significantly reduced gene expression of cytochrome-C (CYC1), mitochondrial transcription factor A (TFAM), mitochondrial membrane ATP synthase (mtATP6), ATP synthase subunit B (ATP5PB), mitochondrial DNA (mtDNA), and caused a slight reduction in nuclear respiratory factor 1 (NRF-1), but no change in ERα. Furthermore, the synergistic treatment did not induce RS production, which may be appropriate for chemotherapy [116].

The molecular interactions of hesperidin extracted from *C. limetta* with the Bcl-2, Bcl-W, myeloid cell leukemia 1 (MCL-1), and ERα receptors overexpressed in breast cancer were investigated. Hesperidin was found to have strong binding energy with BCL-W, MCL-1, and ERα proteins, and the hesperidin-MCL-1 complex was more stable. Following these analyses, hesperidin-loaded nanoliposomes were used to test cytotoxicity in MDA-MB-231 and MCF-10A cell lines. Both encapsulated and isolated hesperidin decreased tumor cell proliferation without causing toxicity to healthy cells. These findings suggest that hesperidin may be a promising target for breast cancer treatment [117].

### 3.5. Induction of Cell Death via Regulation of Apoptotic Signaling Pathways by Naringenin and Hesperidin

Apoptosis is programmed cell death responsible for the balance between proliferation and induction of death. This biological phenomenon replaces senescent, injured, or disease-derived cells. Disrupting the machinery that promotes this cellular control can allow genomic-damaged cells to survive, allowing their uncontrolled proliferation and initiating carcinogenesis. Cancer therapy is based on inhibiting cell proliferation and blocking or stimulating the signaling pathways that lead to the death of these aberrant cells [118]. Therefore, compounds that selectively induce cancer cell death are potential candidates for treating the disease. However, cancer’s high clinical, morphological, and biological heterogeneity makes developing new therapies challenging and time-consuming. Thus, it is critical to understand how new compounds, such as naringenin and hesperidin, interact with cell signaling and how they induce cell death [119,120,121,122].

Anti-cancer activity of naringenin is related to apoptosis, cell cycle signaling and proliferation, and DNA repair mechanisms of cancer cells. Naringenin was tested against the MDA-MB-231 and MCF-10A cell lines and inhibited cell proliferation in a time and concentration-dependent manner in the MDA-MB-231 cell line. Moreover, naringenin was able to promote cell cycle arrest in the G0/G1 phase and increase in sub-G1 (indicative of apoptosis and DNA fragmentation), in addition to inducing apoptosis, with increased caspase 3/7, DNA fragmentation, and reduction of nuclear factor-kB (NF-kB) binding to DNA. To prove these findings, female Wistar rats that received dimethylbenz[α]anthracene (DMBA) (an immunosuppressive agent and inducer of mammary gland tumors) were treated with naringenin for eight days. Naringenin reduced tumor incidence and tumor burden, reduced thiobarbituric acid reactive substances (TBARS), protein carbonyl and nitrate levels, down-regulated superoxide dismutase (SOD) and catalase expression, and up-regulated glutathione reductase (GR) and glutathione peroxidase (GPx) expression. Naringenin also increased markers of mitochondria-mediated apoptosis, including voltage-dependent anion channel (VDAC) and cytochrome-C (Cyt-C), increasing apoptosis in animals with breast cancer [123].

In another study, pure naringenin and its cyclic aminoethyl derivatives (ND): 4-methyl piperidine (3a), piperidine (3b), morpholine (3c), pyrrolidine (3d), 4 hydroxy piperidine (6-membered ring with -OH group on carbon 4) (3e), 3-methyl piperidine (6-membered ring with methyl group on carbon 3) (3f), thiomorpholine (6-membered ring with sulfur) (3g) and piperazine (6-membered ring with nitrogen) (3h)) were tested against several cell lines, including MCF-7, to assess viability and toxicity. The authors observed that 3a–3d reduced the proliferation of the tumor cell lines without causing damage to healthy cells. The compounds 3e–3h were highly cytotoxic. From these data, pure naringenin and ND 3a–3d were tested for their anticarcinogenic effects. After treatment, induction of selective apoptotic cell death was observed in MCF-7 by targeting intrinsic apoptosis signaling pathways and increased expression of p53, which was related to increased expression of Bax and suppression of Bcl-2 gene expression. There is a relationship between Bax/Bcl-2, in which Bax is favored due to Cyt-C and Apaf-1 (apoptotic protease activation factor 1). An increase in these factors was demonstrated when compared to the control group. The overexpression of these proteins forms the apoptosome (protein complex) in the cytosol, leading to an increase in caspase 3, which is responsible for apoptosis [124].

Naringenin was found to decrease the metabolic activity and the number of colony formations in MDA-MB-231 and MCF-7 breast cancer cells, as well as increase cytoplasmic membrane permeability and induce morphological changes indicating apoptotic cell death [125,126]. It also led to cell cycle arrest and reduced cellular capacity for migration and invasion. In MDA-MB-231 cells, naringenin increased the quantification of caspases 3, 8, 9, and Bax, while Bcl-2 was decreased [125]. In MCF-7 cells, naringenin reduced the phosphorylation of histone H3 (pH3), resulting in G2/M cell cycle arrest and increased the activities of poly (ADP-ribose) polymerase (PARP) and caspases 3 and 9, leading to an increase in the number of apoptotic cells [126].

Cyclin-dependent protein kinase 6 (CDK6) is overexpressed in many types of cancer and is responsible for regulating multiple pathways that maintain cell growth and development. Naringenin was also described to be strongly bound to CDK6, thereby preventing tumor development and progression in A549 and MCF-7 cells. Naringenin interacts with CDK6, leading to decreased viability of MCF-7 cells, inducing apoptosis, and reducing the ability to form colonies. This suggests that naringenin may act as a CDK6 inhibitor and can further direct future therapeutic approaches [127].

Naringenin also acts as an adjuvant in breast cancer [108,128]. MDA-MB-231 and MCF-10A treated with pure naringenin (NGEN) and naringenin complexed with copper (Cu(II)) and 2,2′-bipyridine (NGENCuB) were tested. The study exhibited its antiproliferative effect on MDA-MB-231 cells treated with NGEN and NGENCuB. Moreover, the co-treatment was also able to alter morphology, decrease wound closure and the number of colonies, and, in addition, showed apoptotic nuclei with up-regulation of caspase-9 expression. However, NGEN and NGENCuB reduced the viability of the normal lineage by 10% and 30%, respectively [129].

Similarly, the association of naringenin with doxorubicin and metformin affected the cell proliferation of MDA-MB-231 and 4T1 (mammary gland cancer) cell lines, being more effective in reducing cell proliferation, particularly in the 4T1 cell line. In the same study, breast carcinoma was chemically induced and treated with naringenin, liposomal doxorubicin (lipo-dox), and metformin separately or in combination for 28 days. The treatments led to a reduction in tumor weight and an increase in the necrotic area without any effect on blood glucose levels, body weight, or survival. The same results were observed when mice with orthotopic 4T1-induced breast carcinoma were treated with naringenin, metformin, and lipo-dox [128].

The effects of the flavonoids naringenin, quercetin, and naringin, alone or in combination with the type 1 ribosome-inactivating protein, balsamin, on HepG2 (human hepatocarcinoma) and MCF-7 cell lines were evaluated. Treatment with naringenin, quercetin, and naringin together with balsamin reduced the viability of HepG2 and MCF-7 cells, increased caspase-3 and -8 activation, and induced apoptosis through the up-regulation of Bax (BCL-2 associated X protein), Bid (BH3 interacting domain death agonist), Bad (BCL2 associated agonist of cell death), and p53 gene and down-regulation of Bcl-2 and Bcl-XL. These effects were most effective in both cells’ balsamin-naringenin and balsamin-quercetin combinations. Furthermore, the co-treatments were also able to increase the expression of the glucose-regulated protein (GRP) 78 and C/-EBP homologous protein (CHOP) (markers of endoplasmic reticulum stress (ERS)) in HepG2 and MCF-7. Therefore, combining flavonoids with balsamin can be a promising therapeutic approach to sensitize cells and enhance efficacy in breast and liver cancer therapy [130].

Hesperidin also shows proven cytotoxic activity in the literature. A study conducted on the synergism of the natural bioflavonoid compound hesperidin ((2S)-3′,5-dihydroxy-4′-methoxy-7-[α-L-rhamnopyrano-syl-(1→6)-β-D-glucopyranosyloxy]flavan-4-one, HSP) found in oranges and lemons with a synthetic derivative (3,5,7,8-tetrahydro-2-4-(trifluoromethyl)phenyl-4H-thiopyrano-4,3-dpyrimidin-4-one, XAV939), to evaluate the cytotoxic potential obtained in molecular and pathological profiles against HepG2 and MDA-MB-231 cell lines, revealed that the cytotoxicity was cell type- and concentration-dependent. HSP-XAV showed IC_50_ 10.25 µg/mL and 17.1 µg /mL for MDA-MB-231 and HepG2 cells, respectively. There was significant upregulation of the phosphoprotein 53 (p53) and pro-apoptotic genes, such as the X protein associated with B-cell lymphoma (Bax, creatine kinase (CK), and Caspase-3). In B-cell lymphoma, the anti-apoptotic gene (Bcl-2) was significantly down-regulated. In addition, the treatment increased RS levels, accompanied by higher DNA accumulation during the G2/M phase in both cell lines. According to the results, the authors suggest that the synergism promoted between HSP and XAV may be promising as an alternative in the therapy of human liver and breast cancer [131].

Changes in the proliferation, apoptosis, and cell cycle of MDA-MB-231 and MCF-7 breast carcinoma cells were compared concerning the effects of flavonoids hesperidin, apigenin, genistein, naringin, and quercetin. Their cytotoxic activity showed that only hesperidin at a lower dose (5 µM) significantly reduced the cell viability of MDA-MB-231 cells and presented the highest cytotoxic activity with a 100 µM dose in MCF-7 cells. Further analysis revealed that unlike all flavonoids tested, hesperidin did not reduce the percentage of live cancer cells or stimulate apoptosis, although increasing the dosage resulted in an increased number of dead cells. Therefore, the cell cycle progression of MDA-MB-231 and MCF-7 changed significantly after treatment with hesperidin, increasing the percentage of cells in phase G0/G1 [132].

The administration of hesperidin and luteolin demonstrated anti-cancer activity against the MCF-7 cell line. It was reported that in a dose-dependent manner, treatment with 100 or 140 mg/mL effectively reduced cell viability in MCF-7 cells to approximately 36% for hesperidin and 15% for luteolin after 48 h, increasing apoptotic cell populations. From these data, treatment with both compounds resulted in cell cycle arrest, accumulating cell population in the sub-G1 phase or the G0/G1 phase. Hesperidin and luteolin-induced apoptosis in MCF-7 cells led to caspase-3 and -9 expression in hesperidin-treated cells and increased expression of both caspase-9 and -8 in luteolin-treated cells, with the expression of miRNA (miR-16, -34a and -21). In contrast, an increase in the expression of pro-apoptotic proteins Bax was observed [133].

P-glycoprotein (P-gp) transporter is one of the main proteins that contribute significantly to the development of MDR [134]. Interestingly, hesperidin has been investigated to overcome doxorubicin resistance in MCF-7-resistant doxorubicin cells (MCF-7/Dox). In response to treatment, hesperidin increased MCF-7/Dox cells’ sensitivity to doxorubicin (IC_50_ value of 11 µmol/L) compared to MCF-7 cells. Thus, combining hesperidin with doxorubicin inhibits cancer cell growth and prevents resistance by suppressing P-gp expression [135]. Another study also investigated the influence of hesperidin and apigenin (API) on doxorubicin-treated MCF-7 breast cancer cells. First, an optimal concentration of apigenin and hesperidin (50 M) was used to sensitize cells in DOX treatment, and the synergistic effects on MCF-7 viability were confirmed.

Moreover, the combination treatment did not inhibit the cell cycle but showed an increase in cells in the subG1 phase, which corresponds to the dead cell population. It was also confirmed that a co-administration of hesperidin and apigenin with doxorubicin reduced the expression of genes involved in DNA repair, which API + Dox reduced the expression of genes (ERCC11, MSH2, MGMT, and XPC) in 70%, and hesperidin + Dox reduced expression of genes (ERCC1, ATM, OGG1) in over 80%. In summary, these flavonoids have shown an ability to enhance the effectiveness of classical anti-cancer drugs [136].

As discussed above, the search for potential targets to inhibit BCSCs using bioinformatics is also related to hesperidin. In one study, a functional network analysis was performed, and 75 likely therapeutic target proteins correlated with hesperidin were identified, with p53 emerging as a critical gene for the inhibition of BCSCs. In vitro experiments showed that hesperidin was cytotoxic to MCF-7 cells, decreased colony formation and migration ability, and induced cell cycle arrest in G0/G1 phase. In addition, hesperidin treatment significantly downregulated MMP-9 and aldehyde dehydrogenase 1 (ALDH1) while up-regulating cyclin D1. Thus hesperidin can be used to develop drugs for BCSCs [137].

A recent study demonstrated the chemopreventive potential of hesperidin alone and in combination with doxorubicin against DMBA-induced breast cancer in female Wistar rats. Animals pretreated with hesperidin showed a decrease in tumor volume and incidence and a significant improvement in survival rate compared with the control group. In this study, an association between antioxidant and anti-inflammatory effects was found, resulting in a substantial decrease in malondialdehyde (MDA) and an increase in the concentration of GSH in the pretreated animals. Also, improvement in the inflammatory response and reduced organ damage and toxicity was found when compared to doxorubicin alone. The expression of the cell proliferation indicator Ki67 was analyzed. It showed that hesperidin is associated with attenuated Ki67 expression, resulting in a slight improvement in tumor spread and invasion [138].

In another study, the pretreatment of male Wistar rats with hesperidin before cisplatin administration resulted in less liver damage when compared to cisplatin alone. Animals with hesperidin pretreatment showed a significant reduction of known parameters induced by cisplatin, such as serum AST and ALT activity, as well as decreased triglycerides and total cholesterol. Oxidative stress markers resultant of cisplatin in the liver, such as MDA and NO metabolites, were also reduced, as opposed to GSH content, which was significantly higher. Cisplatin also activates a proinflammatory cascade, leading to tissue damage. However, prior administration of hesperidin resulted in NF-kB downregulation, ameliorating this inflammatory response and up-regulating p-Akt, a serine/threonine kinase that promotes cell survival and apoptosis blockade. Furthermore, co-administered cisplatin and hesperidin in several concentrations on MCF-7 cells did not differ from the cytotoxic activity of cisplatin alone. Thus, hesperidin demonstrated a protective effect against cisplatin toxicity in rats without affecting cisplatin’s antitumoral effect [139].

Indeed, under in silico, in vitro, and in vivo approaches, naringenin and hesperidin can interfere with or target distinct cellular pathways in breast cancer cells (Table 2). However, many of these mechanisms still need to be fully understood, which may influence clinical outcomes. Because of this, it is interesting to consider further investigations for possible therapeutic applications.

### 3.6. Inhibition of Tumor Invasion and Metastasis by Naringenin and Hesperidin

Metastasis is considered one of the main problems for breast cancer patients, resulting in more than 90% of cancer-related deaths. During the metastatic process, cancer cells escape from the primary tumor, promote migration, adhesion, and invasion in a different location, and may settle predominantly in the bones, lungs, liver, brain, and lymph nodes [140,141].

The signaling transducer and activator of transcription 3 (STAT3) are activated in various types of cancer and are related to cell proliferation, migration, and invasion [142]. Thus, treatment with naringenin in MDA-MB-231 cells showed a decrease in cellular metabolic activity and an increase in apoptosis and its markers, such as Bax, caspase 3, and 9, decreasing the Bcl-2 protein. However, the co-administration of naringenin with cyclophosphamide enhanced the antitumor effect against this cell line. In addition, naringenin also inhibited the IL-6 effect on the Janus-kinase 2/signaling transducer and activator of the transcription 3 (JAK2/STAT3) pathway by blocking STAT3 phosphorylation, consequently decreasing cell proliferation capacity [143].

As mentioned earlier, estrogen metabolism plays a significant role in mediating breast cancer initiation and development; higher plasma levels and more prolonged exposure to estrogen increase the risk for this disease. To examine the modulatory mechanism and effects of naringenin in estrogen metabolism, chronic psychological stresses, which increase circulating estradiol concentration and promote breast cancer growth, were experimentally induced. In zebrafish (WT AB staining) and C57BL/6 female mice models, naringenin decreased psychological stress, reducing estradiol levels, thus limiting breast cancer growth and metastasis [144].

The control of breast cancer invasiveness and growth is also explained by abnormal signaling by TGF-β cytokines. In advanced-stage tumors, TGF-β activity is upregulated, stimulating the secretion of pro-angiogenic factors, extracellular matrix proteins, and suppression of the immune response culminating in epithelial-mesenchymal transition (EMT), reducing cell adhesion and increased motility [145,146]. Interestingly, naringenin as a treatment prevented TGF-β1 secretion from the 4T1 cell line and suppressed pulmonary metastasis. In this study, the role of protein kinase C (PKC) in regulating the intracellular trafficking machinery of TGF-β cytokines from the trans-Golgi network (TGN) compartment to the cell membrane was analyzed. The proposed mechanism was that naringenin decreased TGF-β1 trafficking from the trans-Golgi network via inhibiting PKC phosphorylation or activity, leading to the accumulation of intracellular TGF-β1, which suppressed tumor cell migration. These results suggest that naringenin can achieve antimetastatic activity by developing anti-cytokine therapies [147].

Dietary phytochemicals, such as hesperidin, allicin, and astragalus polysaccharides present in citrus fruits combined with an optimal diet, have inhibitory effects on breast cancer metastasis. For this, Balb/c mice were xenografted with 4T1 (mammary gland cancer) cells to evaluate the development of primary tumors and detect circulating tumor cells (CTCs) on days 7, 14, 21, and 28. The authors observed that the diet interventions inhibited primary tumor growth and metastasis to the lung. When they were combined with the phytochemicals tested, this effect was enhanced. Furthermore, the inhibitory effect of hesperidin on breast cancer metastasis occurred before day 14 and after day 21. Thus, these dietary compounds and dietary patterns can be evaluated as adjuvant therapies in cancer patients [148].

The overexpression of programmed death ligand 1 (PD-L1) is associated with triple-negative breast cancer (TNBC) (highly metastatic). The EMT process mediated PD-L1 upregulation through PI3K/Akt, mothers against decapentaplegic (SMAD), NF-κB, and ERK/MAPK signaling pathways, with consequent cell migration. Moreover, PD-L1 expression is accompanied by immune evasion modulation, resulting in tumor growth [149]. In this sense, hesperidin exhibited in vitro activity against MDA-MB231 cells by decreasing mRNA levels and PD-L1 protein expression by suppressing Akt and NF-κB signaling pathways. In addition, hesperidin reduced the secretion of the matrix metalloproteinases (MMP-9 and MMP-2), inhibiting migration in MDA-MB-231 cells with high PD-L1 expression. Overall, hesperidin acts as an antitumor agent, and immunotherapy targeting PD-L1 can improve treatment efficacy [150].

**Figure 4:**
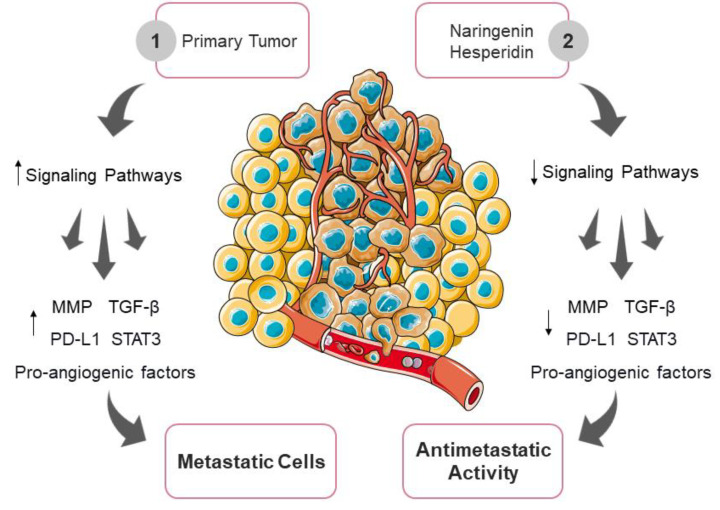
Antimetastatic potential of naringenin and hesperidin. (1) The primary tumor is capable of metastasis under the influence of abnormal signaling pathways, such as increased expression of activator of transcription 3 (STAT3), transforming growth factor-β (TGF-β), pro-angiogenic factors, matrix metalloproteinases, and programmed death ligand 1 (PD-L1); (2) Administration of naringenin and hesperidin inhibited tumor cell migration by blocking these activated signals, which reverse the epithelial-mesenchymal transition (EMT) process and consequent loss of ability to disseminate.

Doxorubicin is essential to breast cancer chemotherapy; however, long-term use causes EMT and initiates invasion through lamellipodia formation, a fundamental first stage of the metastatic process [151]. Thus, the effect of *Citrus sinensis* (L.) peel extract (CSP) in combination with doxorubicin on the MDA-MB-231 cell line was examined. The CSP extract containing hesperidin and naringenin increased cytotoxicity and inhibited the induction of metastasis in these cell lines, suggesting that CSP is a potential co-chemotherapy agent to be developed (Figure 4) [152].

### 3.7. Nanotechnology as a Potentiator of Naringenin and Hesperidin Activity

Nanotechnology has been increasingly used in drug development as it improves bioavailability and produces co-delivery of two or more drugs. There are several nanoparticle delivery systems that can be employed for this purpose. Polymeric nanoparticles, such as polymeric micelles, dendrimers, nanogels, and nanocapsules, are considered to be nanocarriers made of biodegradable polymers. Their preparation can be done in nanospheres or nanocapsules, where the nanosphere is encapsulated uniformly within the polymer chains, and in the nanosphere, the drug is placed in the center and surrounded by a polymeric membrane. Lipid-based nanoparticles can be made of solid lipids or solid and liquid lipids. The main lipids in the nanoparticle are free fatty acids, phospholipids, glycolipids and sphingolipids, steroids, waxes, and triglycerides. Nanosuspensions cause the appearance of particles with a size <1 µm, which are drug-release systems that contain a pure therapeutic agent and a stabilizer. It can be a good choice to solve the low bioavailability and pharmacokinetics of insoluble drugs. Nanoemulsions are prepared by combining surfactants, oils, hydrophilic solvents, and co-solvents that have the unique ability to form fine colloidal dispersions of oil in water [153,154,155,156,157]. Therefore, the use of nanotechnology offers a considerable advantage to the pharmacological potential of flavonoids, which have low solubility, rapid metabolism, and poor absorption in the gastrointestinal tract [156]. In this regard, the citrus compounds naringenin and hesperidin were nano-encapsulated and studied for their potential for successful drug delivery and promising results.

Hesperidin was synthesized by a nanoprecipitation technique using Poly (D, L-lactic-co-glycolic acid) (PLGA) polymers and Poloxamer 407 (a stabilizer) and tested on the MCF-7 cell line to increase stability and bioactive potentials. After treatment, nanohesperidin reduced proliferation and colony formation and induced apoptotic cell death, with increased expression of p53 and caspase-3, compared to native hesperidin. Moreover, nanohesperidin promoted DNA fragmentation. Finally, when tested against human erythrocytes, the modified hesperidin did not cause hemolysis. Therefore, hesperidin nanoparticles have the potential to be developed as a chemotherapeutic agent for human breast cancer, but further investigation is required [158].

In another study, hesperidin was synthesized using a chemical synthesis technique, loaded onto gold nanoparticles (Hsp-AuNPs), and tested on MDA-MB-231 and HBL-100 (normal human breast epithelial) cell lines. It was observed that the synthesized Hsp-AuNPs exhibited higher anti-cancer activity compared to hesperidin or AuNPs separately, without causing damage to normal cells. In the crystal violet assay (also used in cytotoxicity evaluation), Hsp-AuNPs induced morphological changes in tumor cells, including impaired cell-cell communication and reduced cell clusters. Normal cells maintained their full morphology. Furthermore, Hsp-AuNPs promoted the induction of cell death through apoptotic mechanisms. To confirm these data, male Balb/c mice were treated with a Hsp-AuNP dose ranging from 20–200 µg kg for 14 days and then their body weight and cytotoxicity in the kidney and liver were analyzed. Hsp-AuNPs did not alter serum concentrations of alanine transaminase (ALT), aspartate transaminase (AST), or alkaline phosphatase (ALP). In assessing tumor growth in Ehrlich tumor-bearing mice, it was observed that Hsp-AuNPs inhibited growth by inducing functional macrophage activity. In addition, pro-inflammatory cytokines (IL-1β, IL-6, and TNF-ɑ) derived from bone marrow macrophages were inhibited after treatment with Hsp-AuNPs, demonstrating its antioxidant activity. Finally, in the human erythrocyte hemolysis assay, it was shown that synthesized Hsp-AuNPs are potentially biocompatible and can be safely used within the body [159]. Thus, Hsp-AuNPs may be effectively used in clinical cancer therapy and explored for drug delivery applications.

Although exhibiting therapeutic effects, naringenin is a hydrophobic compound with low oral bioavailability [160]. Thus, dextran-coated magnetic nanoparticles loaded with curcumin-naringenin (CUR-NAR-D-MNPs) were prepared by chemical coprecipitation and tested on MCF-7 cells. It was observed that MCF-7 cells treated with CUR-NAR-D-MNPs had reduced proliferation and were induced to die by apoptosis after 48 h of incubation. The co-treatment, using CUR-NAR-D-MNPs and single dose 6 Gy radiotherapy (represents the amount of ionizing radiation energy absorbed) on the tumor cells, caused apoptotic and necrotic cell death and increased RS levels. However, cells incubated with CUR-NAR-D-MNPs 48 h before radiotherapy had exacerbated apoptosis and necrosis percentages compared to those that received radiotherapy, indicating this compound’s antiproliferative and radiosensitizing activity. When evaluating the effect of CUR-NAR-D-MNPs in female Sprague Dawley rats, the treatment reduced tumor volume, leading to cell cycle arrest and induction of apoptosis through modulation of signaling, high p53, high p21, low TNF-α, low CD44, and high RS [161].

Naringenin nanosuspension (NARNS) was prepared using a high-pressure homogenization method with polyethylene glycol D-α-tocopheryl succinate 1000 (TPGS) as a co-stabilizer. This study evaluated the ability of TPGS-coated NARNS to reverse drug resistance in the MCF-7 cell line and human breast adenocarcinoma animal model. In vitro, NARNS demonstrated greater cytotoxic efficacy when compared to free NARNS. The treatment reduced GSH levels and increased mitochondrial membrane potential, intracellular RS, lipid peroxidation (TBARS), and caspase-3 activity, also showing apoptotic index (membrane blebs and nuclear fragmentation). In the animal model of breast adenocarcinoma, mice treated with NARNS exhibited a decrease in the number of tumor cells and a longer life expectancy. Therefore, NARNS can be considered a good chemotherapeutic agent [162].

Flavonoids such as naringenin and hesperidin have already demonstrated health benefits and positive results against cancer, but poor absorption is still a problem. Nanoparticles in this scenario are a promising target as they are a technology that improves the delivery of compounds.

## 4. Conclusions

Citrus fruits commonly present in the human diet are one of the most important dietary sources of flavonoids, and naringenin and hesperidin have significant impacts on many biological processes. The rising prevalence of breast cancer, whose primary therapeutic approach is represented by chemotherapy with side effects and resistance, brings to light the discussion of the role of natural antioxidants as possible co-adjuvant therapeutic agents. Managing cancer therapy to improve efficacy involves a more detailed explanation of molecular targets and signaling pathways by increasing the selectivity for cancer cells. The main mechanisms of hesperidin and naringenin in breast cancer are linked to their ability to interfere with cell survival by inhibiting proliferation and reducing tumor growth, volume, and incidence. These compounds also play a direct role in modulating epigenetic and estrogen receptor activity. In the case of cell death and metastasis, administration of these flavanones may induce apoptosis and impair the ability to metastasize. Based on the anticancer effects of flavanones, it is clear that further efforts are needed to treat patients and that many important aspects still need to be explored to improve our understanding of these compounds in cancer.

## Figures and Tables

**Table 1:** Activities of several citrus-derived natural bioactive compounds.

Compounds	Classification	Review Highlights
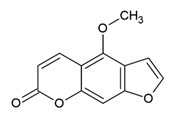 Bergapten 5-Methoxypsoralen	Polyphenol class: Other polyphenols Polyphenol sub-class: Furanocoumarins Family: Furanocoumarins	Anti-inflammatory, antimicrobial, antifungal, antiviral, anticancer, and antiosteoporosis [52]. Neuroprotection activity, effect on vitiligo and psoriasis, analgesic activity, immunosuppressive properties, and antidiabetics [53].
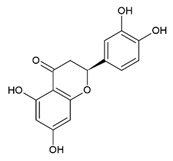 Eriodictyol 5,7,3′,4′-Tetrahydroxyflavanone	Polyphenol class: Flavonoids Polyphenol sub-class: Flavanones Family: Flavanones	Antioxidant, anti-inflammatory, anticancer, neuroprotective, cardioprotective, hepatoprotective, anti-diabetic, and anti-obesity activity [50]. Skin protection, immunomodulatory, analgesic, antipyretic, antinociceptive, and miscellaneous activities [51].
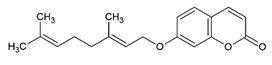 Auraptene 7-geranyloxycoumarin	Class: Phenol lipids Sub-class: Terpene Lactones Family: Terpene Lactones	Antitumor activity against BC, colorectal, ovarian, skin, gastric, esophageal, hepatic, and prostate cancer [56]. Cardioprotective, gastrointestinal protective, immune protective, and miscellaneous effects [57]. Effects on neurodegenerative diseases, periodontal disease, oncogenesis, cystic fibrosis, hypertension, and lipid profile [58].
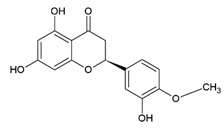 Hesperetin 5,7,3′-Trihydroxy-4′-methoxyflavanone	Polyphenol class: Flavonoids Polyphenol sub-class: Flavanones Family: Methoxyflavanones	Antioxidant and anti-inflammatory effects [45]. Anticancer activities against glioblastoma, breast, lung, prostate, colon, liver, pancreatic, kidney, gastric, oral, ovarian, and leukemia [46].
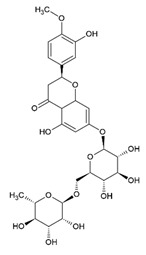 Hesperidin Hesperetin 7-*O*-rutinoside	Polyphenol class: Flavonoids Polyphenol sub-class: Flavanones Family: Flavanones	Effects on cardiovascular, neurological, psychiatric disorders, and antitumor activity [42]. Lipid metabolism, glucose metabolism, and inflammation activity [43]. Improvements in epidermal permeability barrier function, protection against UV irradiation, melanogenesis, acceleration of cutaneous wound healing, antioxidant [44].
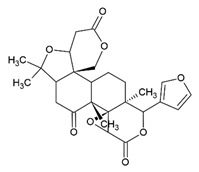 Limonin	Class: Prenol lipids Sub-class: Triterpenoids	Anticancer, anti-inflammatory, analgesic, antibacterial, antiviral, anti-insect, antioxidant, liver protection, neuroprotection, anti-osteoporosis, anti-obesity, and anti-allergy activities [59].
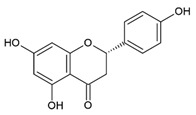 Naringenin 5,7,4′-Trihydroxyflavanone	Polyphenol class: Flavonoids Polyphenol sub-class: Flavanones Family: Flavanones	Immunomodulator [60], neuroprotective effects [61], anticancer activity against breast, colorectal, lung, liver, brain, leukemia, lymphoma, skin, cervical, prostate, pancreatic, gastric, oral, osteosarcoma, bladder, and ovarian cancer [62]. Effects on atherosclerosis, coronary artery disease, hypertension, cardiac hypertrophy, myocardial infarction, ischemic stroke [63], and fibrosis [64]. Antioxidant, antiviral, and antidiabetic [65].
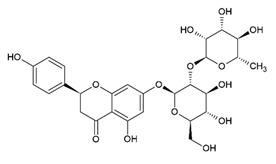 Naringin Naringenin 7-*O*-neohesperidoside	Polyphenol class: Flavonoids Polyphenol sub-class: Flavanones Family: Flavanones	Effect on obesity, diabetes, hypertension, cardiac toxicity, hypertrophy, remodeling, steatosis, hepatic protection, atherosclerosis, oxidative stress [66], metabolic syndrome, bone regeneration, genetic damage, central nervous system (CNS) diseases, anticancer and anti-inflammatory activity [67,68].
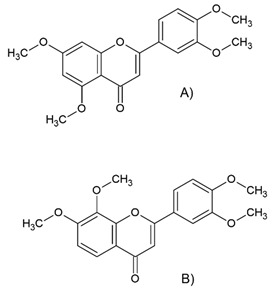 (A) 5,7,3′,4′-Tetra-methoxyflavone (B) 7,8,3′,4′-Tetra-methoxyflavone	Polyphenol class: Other polyphenols Polyphenol sub-class: Polymethoxyflavones Family: Polymethoxyflavones	Effects on circadian rhythm and metabolism [47] and neurodegenerative diseases [48]. Induces apoptosis via modulating the anti-tumor immunity, endoplasmic reticulum (ER) stress-mediated apoptosis, epigenetics modulators, protective autophagy, pyroptosis, ferroptosis, and anoikis cell death [49].
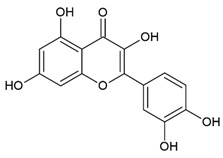 Quercetin 3,5,7,3′,4′-Pentahydroxyflavone	Polyphenol class: Flavonoids Polyphenol sub-class: Flavanols Family: Flavonols	Anti-cancer properties in BC, prostate cancer, ovarian cancer, lung cancer, colon cancer, hepatocellular carcinoma, lymphoma, and pancreatic cancer [15]. Effects on autoimmune diseases [35], metabolic syndrome [36], oxidative stress, and autophagy [37]. Anti-allergic [38], anti-inflammatory, anti-hypertensive [39], antiviral [40], and neuroprotective efficacy [41].
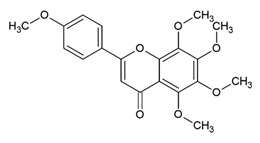 Tangeretin 5,6,7,8,4′-Pentamethoxyflavone	Polyphenol class: Flavonoids Polyphenol sub-class: Flavanes Family: methoxyflavones	Antitumor, neuroprotective, antidiabetic, hepatoprotective, immunomodulatory, melanogenesis, and antioxidant activities [54]. Induces apoptosis and autophagy and suppresses migration, invasion, and angiogenesis [55].

Structures presented in Table 1 were obtained from the ACD/ChemSketch software (Freeware) based on those presented in the original articles.

**Table 2:** Summary of the mechanisms of naringenin and hesperidin in different analyses.

Compounds	Type of Study	Experimental Aspects	Proposed Mechanism	Reference
Naringenin	In vitro and in vivo	MDA-MB-231 and MCF-10A cell lines and female Wistar rats (120–160 g)	↓cell proliferation, tumor incidence and weight, TBARS, SOD, catalase, protein carbonyl, nitrate, GSH, vitamin C, vitamin E, GR, Bax, and Bad, ↑body weight (DMBA group) ↑G0/G1 and sub-G1 cell cycle, ↑caspase-3/-7, Apaf-1, VDAC, Bcl-2, cytochrome c, Bcl-xl, and procaspase-9	[123]
	In vitro	MCF-7, HT29, HeLa, DU145, and C8-D1A cell lines	For MCF-7: ↓cell proliferation, ↑expression P53 gene, Bax, cytochrome c, Apaf-1, and caspase-3	[124]
	In vitro	MDA-MB-231 cell line	↓cell proliferation, migration, invasion, and colony formation, ↑apoptosis, caspases-3/-8/-9, Bax, and ↓Bcl-2, ↑G2/M cell cycle	[125]
	In vitro	MDA-MB-231 cell line	↓cell viability, colony formation, percentage of pH3-positive cells, and ↑apoptosis, caspase-3/-9, anti-PARP levels, LDH release and G2/M cell cycle	[126]
	In silico and in vitro	MCF-7 and A549 cell lines	↓cell viability and colony formation, ↑apoptosis and binding affinity to CDK6	[127]
	In vitro	MDA-MB-231 and MCF-10A cell lines	↓cell proliferation, migration, colony number and size, pro-MMP9 activity, and ↑induce apoptosis/necrosis	[129]
	In vivo	Female Sprague Dawley rats (80–120 g) and female Balb/c mice (18–22 g)	↓tumor weight, volume, and ↑tumor necrosis	[128]
	In vitro	MCF-7 and HepG2 cell lines	↓cell viability, ↑apoptosis, caspase-3/-8, Bax, Bid, Bad, p53, ↑GRP78 and CHOP	[130]
Hesperidin	In vitro	MDA-MB-231 and HepG2 cell lines	↓cell viability, ↑caspase-3, Bax, and p53, ↓Bcl-2, ↓MMP1, ↑ROS, ↑G2/M cell cycle, apoptotic and nuclear fragmentation	[131]
	In vitro	MCF-7 and MDA-MB-231 cell lines	↓cell viability, cell cycle arrest, and ↑apoptosis	[132]
	In vitro	MCF-7 and HEK 293 cell lines	↓cell viability, ↑number of apoptotic cells, ↑G0/G1 and sub-G1 cell cycle, ↑caspase-3/-9, ↑miR-16 and -34a, ↓miR-21, ↑Bax and ↓Bcl-2	[133]
	In vitro	MCF-7-resistant doxorubicin cells (MCF-7/Dox)	↓cell viability and expression of Pgp	[135]
	In vitro	MCF-7 cell line	↓cell viability, ↑cells in sub-G1 phase, ↑early apoptosis, ↓GSH, ↑DNA damage, ↓expression of DNA repair genes	[136]
	In vitro	MCF-7 breast cancer cell line	↓cell viability, mammosphere formation, colony formation, cell migration, ↑G0/G1 cell cycle, ↓p21, ↑cyclin D1, ↓*ALDH1*, ↓*MMP9,* ↑p53, and ↓Bcl-2	[137]
	In vivo	Female Wistar rats	↑Survival rate, ↑body weight, ↓tumor volume, tumor spread and invasion, ↓MDA, ↑GSH, ↑IL-1β, ↓IL-6, NF-κB, TNF-α, and Ki67 expression	[138]
	In vivo	Adult male Wistar rats (120–150 g)	↓ALT, AST, TG, TC and MDA, ↑GSH, ↓hepatic NO, ↓NF-κB, ↑p-Akt expression	[139]

Abbreviations: DMBA: 7,12-dimethylbenz[a] anthracene; TBARS: thiobarbituric acid-reactive substances; SOD: superoxide dismutase; GSH: reduced glutathione; GR: glutathione reductase; Apaf-1: apoptotic protease activating factor-1; VDAC: voltage-dependent anion channel; Bcl-2: B-cell lymphoma 2; Bcl-xl: B-cell lymphoma-extra large; Bax: Bcl-2 associated X-protein; Bad: Bcl-2 associated agonist of cell death; HT29: colorectal adenocarcinoma; HeLa: cervix carcinoma; DU145: prostate carcinoma; C8-D1A: normal brain astrocyte; LDH: lactate dehydrogenase; pH3: phospho-histone H3; PARP: poli ADP-ribose polymerase; A549: lung adenocarcinoma; NAG: naringenin; CDK6: cyclin dependent kinase 6; pro-MMP9: pro-matrix metallopeptidase 9; HepG2: human hepatocellular carcinoma; GRP78: glucose-regulated protein 78; CHOP: CCAAT/enhancer-binding protein homologous protein; MMP1: matrix metallopeptidase 1; ROS: reactive oxygen species; HEK293: human embryotic kidney 293; miR-16,-34a,-21: microRNAs; Pgp: P-glycoprotein; ALDH1: aldehyde dehydrogenase 1; MDA: malondialdehyde; IL-1β: interleukin 1 beta; IL-6: interleukin 6; NF-κB: nuclear factor kappa-light-chain-enhancer of activated B cells; TNF-α: tumor necrosis factor alpha; Ki67: nuclear antigen; ALT: alanine aminotransferase; AST: aspartate aminotransferase; TG: triglycerides; TC: total cholesterol; NO: nitrate/nitrite; p-Akt: protein kinase B. The arrows represent up-regulated (↑) or down-regulated (↓).

## Data Availability

Not applicable.
